# Fabrication and Characterization of Effective Biochar Biosorbent Derived from Agricultural Waste to Remove Cationic Dyes from Wastewater

**DOI:** 10.3390/polym14132587

**Published:** 2022-06-26

**Authors:** Asmaa Elsherbeny Moharm, Gamal A. El Naeem, Hesham M. A. Soliman, Ahmed I. Abd-Elhamid, Ali A. El-Bardan, Taher S. Kassem, AbdElAziz A. Nayl, Stefan Bräse

**Affiliations:** 1 Department of Chemistry, Faculty of Science, Alexandria University, P.O. Box 426, Alexandria 21321, Egypt; asmaa.moharm1317@gmail.com (A.E.M.); alyelbardan@yahoo.com (A.A.E.-B.); taherkasem@gmail.com (T.S.K.); 2 Advanced Technology and New Materials Research Institute (ATNMRI), City of Scientific Research and Technological Applications (SRTA-City), P.O. Box 179, New Borg AlArab 21934, Alexandria, Egypt; jimmynaeem@yahoo.co.uk (G.A.E.N.); h.soliman@srtacity.sci.eg (H.M.A.S.); ahm_ch_ibr@yahoo.com (A.I.A.-E.); 3 Department of Chemistry, College of Science, Jouf University, Sakaka 72341, Saudi Arabia; 4 Institute of Organic Chemistry (IOC), Karlsruhe Institute of Technology (KIT), Fritz-Haber-Weg 6, 76133 Karlsruhe, Germany; 5 Institute of Biological and Chemical Systems-Functional Molecular Systems (IBCS-FMS), Director Hermann-von-Helmholtz-Platz 1, 76344 Eggenstein-Leopoldshafen, Germany

**Keywords:** biochar, agricultural waste, cationic dyes, wastewater, sugarcane bagasse

## Abstract

The main aim of this work is to treat sugarcane bagasse agricultural waste and prepare an efficient, promising, and eco-friendly adsorbent material. Biochar is an example of such a material, and it is an extremely versatile and eco-friendly biosorbent to treat wastewater. Crystal violet (CV)-dye and methylene blue (MB)-dye species are examples of serious organic pollutants. Herein, biochar was prepared firstly from sugarcane bagasse (SCB), and then a biochar biosorbent was synthesized through pyrolysis and surface activation with NaOH. SEM, TEM, FTIR, Raman, surface area, XRD, and EDX were used to characterize the investigated materials. The reuse of such waste materials is considered eco-friendly in nature. After that, the adsorption of MB and CV-species from synthetically prepared wastewater using treated biochar was investigated under various conditions. To demonstrate the study’s effectiveness, it was attempted to achieve optimum effectiveness at an optimum level by working with time, adsorbent dose, dye concentration, NaCl, pH, and temperature. The number of adsorbed dyes reduced as the dye concentrations increased and marginally decreased with NaCl but increased with the adsorbent dosage, pH, and temperature of the solution increased. Furthermore, it climbed for around 15 min before reaching equilibrium, indicating that all pores were almost full. Under the optimum condition, the removal perecentages of both MB and CV-dyes were ≥98%. The obtained equilibrium data was represented by Langmuir and Freundlich isotherm models. Additionally, the thermodynamic parameters were examined at various temperatures. The results illustrated that the Langmuir isotherm was utilized to explain the experimental adsorption processes with maximum adsorption capacities of MB and CV-dyes were 114.42 and 99.50 mgg^−1^, respectively. The kinetic data were estimated by pseudo-first and pseudo-second-order equations. The best correlation coefficients of the investigated adsorption processes were described by the pseudo-second-order kinetic model. Finally, the data obtained were compared with some works published during the last four years.

## 1. Introduction

The development of various industrial operations in recent decades has resulted in serious environmental pollution by hazardous organic and inorganic materials. Dyes are examples of such dangerous colored ionized aromatic organic pollutants which are utilized in various industries, including textiles, paint, printing, food, paper, leather, wood, cosmetics, and plastics [1,2]. These pollutants have various effects of poisoning, carcinogenic, mutagenic, and posing a health risk [3]. Additionally, the effluents of such wastes may contain various dyes, including cationic dyes, anionic dyes, and azo dyes. MB is a thiazine dye that has the chemical formula C_16_H_18_N_3_ClS. Due to its chemical structure, it has chemical stability and water solubility, making it useful in various industries [4]. The presence of this dye in wastewater can induce eye burns, difficulty or quick breathing, mental confusion, nausea, vomiting, and profuse sweating [5]. CV is a cationic triphenylmethane dye with the chemical formula C_25_H_30_ClN_3_ used as a biological stain, textile colorant, and paper dye. CV can cause respiratory and renal failure, skin and digestive tract irritation, and mammalian cell toxicity when present [6]. Therefore, decolorization and treatment of textile dye wastewaters are very necessary. Different technologies were investigated to remove various pollutants from wastewater, such as reverse osmosis, membrane filtration, chemical precipitation, solvent extraction, biological treatment, oxidation, chemical coagulation/flocculation, irradiation, ion exchange, ozonation, and adsorption [1,7,8,9,10], in addition to other approaches largely used to remove dyes from aqueous media such as catalysis, chemical reductions, and photo-catalysis [7,8]. Adsorption is a potential technology for transferring pollutants from the solvent to the solid phase because of its high efficiency, availability, effectiveness, simplicity, lack of toxic exhaust, and wide accessibility [1,8,9]. Adsorption happens on the pore walls inside the particles, usually porous solids. Activated carbon (which primarily adsorbs organics), activated alumina (which absorbs moisture), silica gel, molecular sieves, synthetic resins, zeolites, and chitosan composites are examples of adsorbents [6]. There is a growing demand for efficient, low-cost, effective, natural, and eco-friendly adsorbents made from alternate sources. Activated carbon is a natural and effective adsorbent that can be prepared from different types of agricultural wastes such as sugarcane bagasse (SCB), wheat straw, rice husk, pine cone, coffee husk, coconut husk, cornstalk, banana, and orange peels. SCB is one of the most world’s productive crop wastes, with a huge output, cheap cost, a concentrated producing region, and stable and uniform qualities that are beneficial in industrial production. SCB is a crystalline cellulose nanofiber embedded in an amorphous matrix of cross-linked hemicellulose and lignin from sugarcane [11,12]. Additionally, it has higher carbon and oxygen contents with lower ash levels than other agricultural wastes, and it is considered an ideal material for preparing carbon products. On the other hand, biochar is a carbon-rich solid substance created by thermally converting waste biomass under anaerobic or low-oxygen conditions [13]. Biochar is mostly composed of carbon, but it also includes oxygen, hydrogen, ash, and trace amounts of sulfur and nitrogen [14]. Biochar stands out among other adsorbents because of its plentiful sources, inexpensive cost, consistent chemical and physical properties, general use, and high recycling rate. Livestock dung, agricultural waste, ocean garbage, and city waste can be used to make it. Biochar is a major adsorbent for hydrophobic organic pollutants due to its aromaticity, large surface area, and surface functions [15]. Burning crop residues such as bagasse, rice, and wheat straw have been found to contribute an additional source of greenhouse gases (GHG) and raise levels of suspended particle matter (SPM), perhaps causing respiratory problems in the environment. Thus, using waste biomass as agricultural waste to produce carbonaceous chemicals can help mitigate the environmental effects of crop waste burning. Converting agricultural waste to biochar, which contains 43%, 26%, and 16% of cellulose, hemicellulose, and lignin could be one solution to reducing greenhouse gas emissions. The composition of biomass and the carbonization method are two aspects that influence the manufacture of the surface of biochar. Pyrolysis and gasification are two common processes for converting biomass to biochar [16]. Many extensively utilized biochar modification procedures, such as alkalinity and/or acid, metal salts, oxidizing agent, and steam and gas purge modification, have improved its sorption capacity for target pollutants [17]. The alkali activation process increases biochar’s activated surface area and adsorption capacities by increasing porosity and clearing partially clogged pores [18]. Alkali activation (mainly KOH and NaOH) is necessary for the following reasons: (i) to fully expose active adsorption sites, chemical activation, physical activation, and metallic intercalation work together to increase the surface area of biochar; (ii) the interaction between the carbon-oxygen bond and the alkaline ion increases functional groups (mostly hydroxyl groups); (iii) to enhance metal ion precipitation and increase surface basicity. NaOH is a cost-effective, more ecologically friendly alternative to KOH that produces great recovery and promotes many functional groups (mostly -OH) via carbon-oxygen bonds and alkaline ionization [19], which are the most advantageous properties of NaOH. Phosphoric acid and Zinc chloride are two of the most often used activating agents in chemical activation, aside from NaOH and KOH [20,21]. Therefore, many research studies have been published during the last couple of years investigating the use of biochar to adsorb dyes and in wastewater treatment processes [22,23,24,25,26,27,28,29,30,31,32,33]. CV and/or MB dyes were adsorbed from aqueous media by various types of biochar such as eucalyptus camaldulensis biochar (Ec-bio) [22], porous biochar [23], mesoporous seaweed biochar [24], castor biomass-based biochar [25], adsorbents from orange peel residues [26], biochar at different carbonization times [28], date palm fronds biochars [31], and rice husk-mediated magnetic biochar (RH-MBC) [33].

Herein, our work was directed towards treating large quantities of sugarcane bagasse agriculture waste and using this strategically in the industry to eliminate the CO_2_ emission that could be produced from burning these wastes. Additionally, another objective of this research was to target the proper valorization of the modified sugarcane bagasse in wastewater treatment. Therefore, this research aimed to prepare an efficient adsorbent through pyrolysis and surface activation with NaOH to remove organic pollutants from wastewater. This work successfully transformed sugarcane bagasse into a carbonous material through a simple preparation strategy. The prepared material can effectively adsorb cationic organic pollutants within a highly short contact time. Further future works are still needed to treat such wastes by eco-friendly methods to acquire maximum benefit.

In this work, biochar was prepared firstly from sugarcane bagasse agriculture waste, and then a novel biochar material was synthesized through pyrolysis and surface activation with NaOH. The prepared material was characterized and investigated to remove cationic dyes (CV and MB) from prepared synthetic wastewater. Different adsorption batch conditions were examined, such as contact time, initial dye concentrations, adsorbent dosage, pH, temperature, and other adsorption factors. Additionally, the thermodynamics and adsorption efficiencies of the investigated adsorption processes were studied using various isotherm and kinetic models. Finally, a comparison study between adsorption capacities of the prepared biochar and other biochar materials used in the recent four years of published works was investigated.

## 2. Materials and Methods

### 2.1. Materials and Instrumentation

The purity grade of all used chemicals and more detailed information about instruments used in this work are explained in the Supplementary Material file (Sections S1.1 and S1.2).

### 2.2. Preparation of Biochar

Bagasse samples from sugarcane were collected from a local market, dried in an oven at 70 °C for 72 h, chopped into small pieces, grounded into a fine powder, and sieved at 1 mm. After that, 1.0 g of sieved fine powder was pyrolyzed in a porcelain crucible in a muffle furnace at 350 °C for 30 min with a 10 °C/min heating rate. The resulting black powder (BC) (0.7 g) was treated with 1% (T-BC-1), 2% (T-BC-2), 5% (T-BC-5), 10% (T-BC-10) NaOH (*w*/*v*) for 24 h with magnetic stirring, then soaked for another 24 h. The finished product was filtered, rinsed several times with distilled water, dried at room temperature, ground to a fine powder, and stored in a sealed container.

### 2.3. Adsorption Experiment, Kinetics, Isotherm, and Thermodynamics Study

Adsorption experiment, kinetics, isotherm, and thermodynamics studies investigated in this work are explained in the Supplementary Material file (Sections S1.3–S1.6, respectively).

## 3. Results and Discussion

### 3.1. Characterization

#### 3.1.1. SEM and TEM Analyses

**Figure 1:**
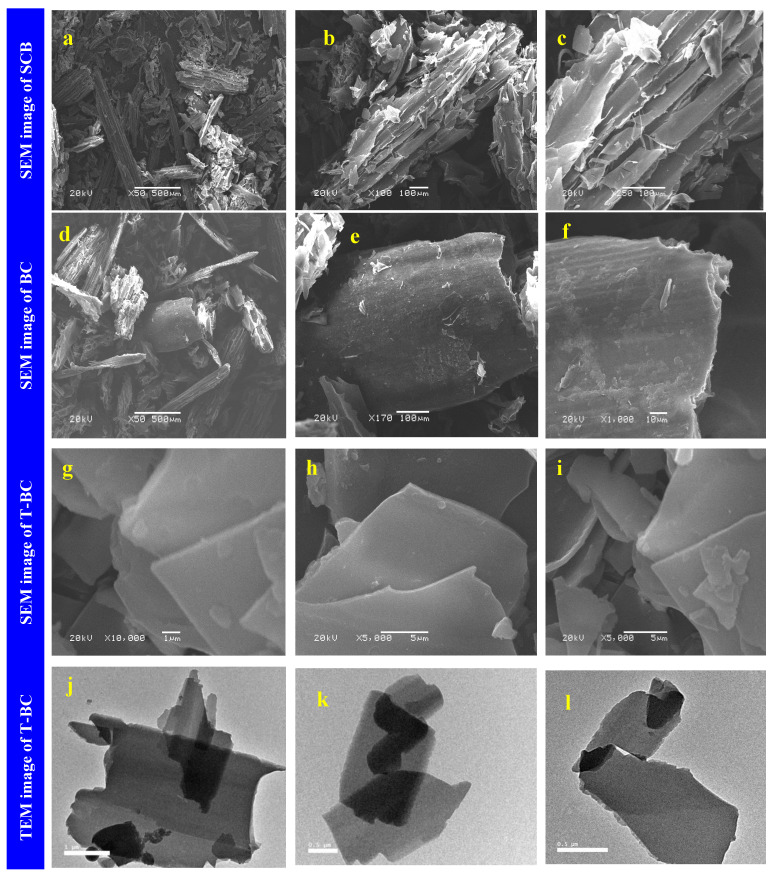
SEM image of SCB (**a**–**c**), BC (**d**–**f**), T-BC (**g**–**i**), and TEM image of T-BC (**j**–**l**).

SCB was thermally treated to form biochar (BC) which was chemically treated with NaOH to compose treated biochar (T-BC). Scanning electron microscope was noted as an efficient device to fellow the surface morphology variation on the SCB before and after the treatment processes, as represented in Figure 1. The SCB powder seemed to have a long, wrinkle rod-like structure. Additionally, SCB showed a porous, fibrous texture, parallel stripes that constituted the fibers, waxes, extractives, and other deposits on the surface with rigid, compact, and thick-walled fiber cells inter-linked with pulp, as shown in Figure 1a–c. After thermal treatment of SCB to form BC, a smooth layered structure with flat surfaces and parallel sheaths was observed. Additionally, the observed flakes have some sheets, as illustrated in Figure 1d–f. This layered structure of BC became more smooth and was ordered after treatment with NaOH to form T-BC. The morphology of each layer became more obvious as the magnification was increased [34], as shown in Figure 1g–i. This ordered layered structure for the treated biochar was further confirmed with the transmission electron microscopy (TEM) technique and represented in Figure 1j–l. The presence of several thin and transparent sheets and a smooth surface was visible and indicated biochar nanosheets’ existence [35].

#### 3.1.2. FTIR Analysis

**Figure 2:**
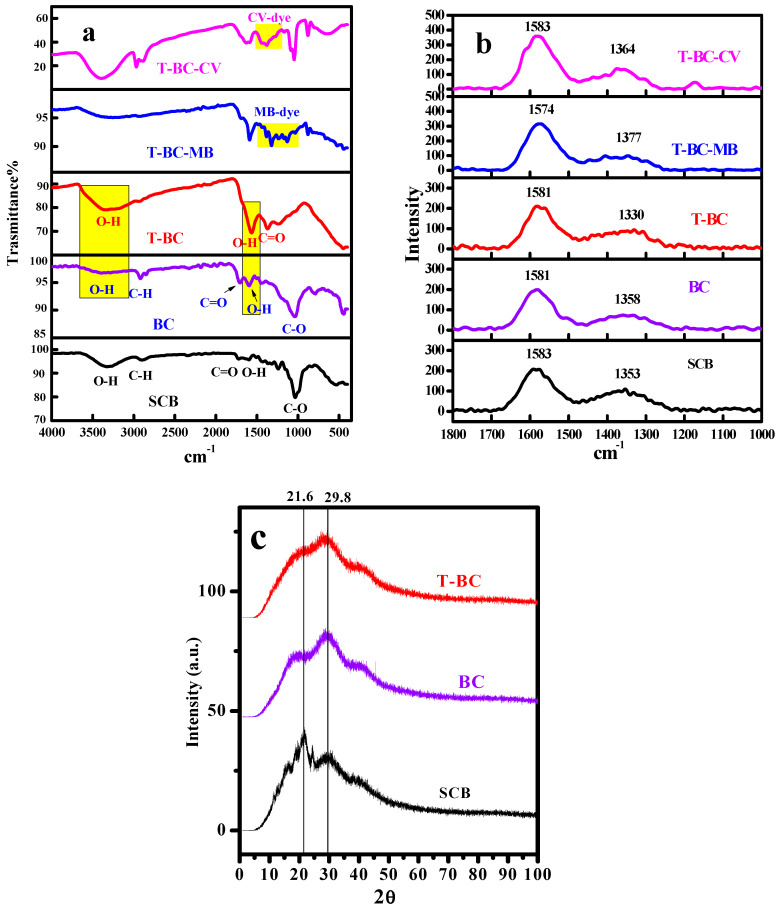
(**a**) FTIR and (**b**) Raman spectra of SCB, BC, T-BC, T-BC-MB, and T-BC-CV, and (**c**) XRD pattern of SCB, BC, and T-BC.

The vibration motion of distinct functional groups of the material surface is determined using FTIR. The resulted spectra of SCB, BC, T-BC, T-BC-MB, and T-BC-CV are shown in Figure 2a. For the SCB spectrum, the bands at 3327 cm^−1^ are due to O-H groups of surface adsorbed water. The two peaks at 2903 and 2349 cm^−1^ are attributed to symmetric and asymmetric vibrations of -CH and -CH_2_. The peak at 1722 cm^−1^ is due to the stretching vibration of C=O of unconjugated hemicellulose and lignin, respectively [36]. The -C=C- and -C=O stretching vibrations of the lignin aromatic ring are responsible for the band at 1599 cm^−1^ [11,37,38], while the band at 1507 cm^−1^ is attributed to lignin ring C=C skeletal vibration, the band at 1427 cm^−1^ belongs to lignin -CH_2_ and -CH_3_ bending, and the peak at 1319 cm^−1^ is due to cellulose -CH_2_ bending [11]. The aryl C–O out of plane stretching vibration of hemicellulose and lignin is responsible for the band at 1241 cm^−1^ [37,38]. The stretching vibrations of -C–O of hemicellulose and cellulose are observed at 1034 cm^−1^ [11,36]. The glycosidic bond β-(1–4) of cellulose has appeared at 831 cm^−1^ [39], and C-H stretching vibrations are represented by the two bands at 831 and 531 cm^−1^ [38,40]. Three peaks, 3871, 3797, and 3397 cm^−1^, are observed due to the presence of O–H stretching vibrations of the C–OH groups and water in the spectra of biochar [18,41]. After thermal treatment of SCB to form BC, the hydroxyl groups of SCB will be condensed into alkoxide groups, reducing the amount of the surface adsorbed. Further treatment of BC with NaOH to compose T-BC, the intensities of the bands related to the water motions at 3332 cm^−1^ and 1565 cm^−1^ will be enhanced, see Figure 2a. This may be attributed to the increased density of oxygenated function groups upon treating the BC with NaOH. This manner will cause T-BC to interact with the solvent from the surrounding environment, strengthening the band’s intensities at 3332 and 1565 cm^−1^. Furthermore, by mixing of T-BC with the dye molecules (MB and CV), the peaks related to the dye species will be observed in the FTIR- spectra. As a result, shifts, disappearance, the appearance of new bands, and changes in peaks were observed after the adsorption processes of dyes species. After MB adsorption, the peak at 3332 cm^−1^ shifted to 3257 cm^−1^, the peak at 1565 cm^−1^ shifted to 1589 cm^−1^, the peak at 1370 cm^−1^ shifted to 1383 cm^−1^, and the peak at 1238 cm^−1^ shifted to 1237 cm^−1^. After CV adsorption, the peak at 3332 cm^−1^ shifted to 3392 cm^−1^, the band at 1565 cm^−1^ shifted to 1630 cm^−1^, and the band at 1370 cm^−1^ shifted to 1380 cm^−1^. Additionally, after adsorption of MB and CV-dyes, new bands at 3480 and 3392 cm^−1^ were observed and attributed to the presence of MB and CV, respectively. In addition to ionic interactions between the -OH groups in T-BC-structure, MB and CV- species are assumed to be the cause of many abrupts decreases, with the band shifting possibly indicating that these functional groups interact with the MB and CV molecules [42].

#### 3.1.3. Raman

Raman is considered an available tool to ascribe the ordered and disordered region’s structure of carbonaceous material. The Raman spectra of SCB, BC, T-BC, T-BC-MB, and T-BC-CV are illustrated in Figure 2b and present two main bands at 1583 cm^−1^ G-band which are attributed to sp^3^ hybridization of carbon atoms and D-band at 1353 cm^−1^ which attributed to hetero atoms and defect sp^2^ hybrid carbon in the carbonaceous lattice [43]. The positions and the ratio of intensities of the two bands **I_D_/I_G_** were listed in Table 1. The intensity ratio of bands D and G (I_D_/I_G_) is important for describing carbon atoms’ disordered structure, including lattice and edge defects caused by sp^3^-C formation due to the treatment process [35,44]. The increase of I_D_/I_G_ indication creation of sp^3^-C, i.e., the addition of hetero O-atoms, affirms the successful treatment process.

#### 3.1.4. Porosity and Surface Area

The surface area, pore volumes, and pore diameters of SCB [45], BC, and T-BC were determined using the BET (Brunauer, Emmett, and Teller) method) and the BJH (Barrett, Joyner, and Halwnda) method, as shown in Figure S1 and Table S1. The samples’ specific surface area was calculated using nitrogen adsorption/desorption isotherms at 77 K, as shown in Figure S1. Nitrogen gas is commonly employed due to its high purity and strong interaction with most substances. From Table S1, in comparing SCB, BC, and T-BC, the T-BC has the greatest values of S_BET_ and Total pore volume, which are 8.2175 (m^2^/g) and 6.3102 (cm^3^/g), respectively. T-BC also possesses a mesoporous structure with an average pore diameter of 30.716 (nm) [43]. This indicates that T-BC may be a better adsorbent for the adsorption processes.

#### 3.1.5. XRD Analysis

XRD was required to detect the crystallinity degree of the tested material. The XRD of SCB, BC, and T-BC are shown in Figure 2c. It can be observed that the SCB sample exhibited broad bands that corresponded to the crystallographic plane of inter-planar spacing of cellulose molecules, with two peaks at (2ϴ ~ 21.646°) and (2ϴ ~ 29.858°), indicating that SCB is amorphous due to the presence of lignin and hemicellulose (amorphous substances) [31,46]. By pyrolysis of SCB at 350° to form BC, the intensity of the peak at (2ϴ = 21.6°) will be weakened while the peak at (2ϴ = 29.8°) will strengthen. This action corresponds to the creation of highly graphitized material [47]. After treating BC with NaOH, the peak (2ϴ = 21.6°) nearly disappeared, and the peak at (2ϴ = 29.8°) became more broadened. The two peaks of modified biochar appeared at (2ϴ ~ 28.418° and 40.335°). This finding suggests the development of highly organized nanosheets-biochar. SCB, biochar, and modified biochar have Crystallinity Index (CI) values of 35.83, 36.81, and 35.29, respectively.

#### 3.1.6. EDX Analysis

EDX was used to detect the elemental composition of the synthesized materials SCB, BC, and T-BC and is represented in Figure S2. It was observed that the SCB is mainly composed of C and O atoms. By pyrolysis SCB at 350 °C, the oxygen functional groups will be condensed and remove water molecules which will reduce the O content in the sample. The Na atoms were observed in the T-BC sample after treating the BC with NaOH.

### 3.2. Adsorption Study

The effect of NaOH concentration on the adsorption efficiency of BC samples ranged from 1.0–10% to form treated-BC and was investigated at various times. Then, the adsorption efficiencies of the prepared T-BC adsorbents were studied and represented in Figure S3. The results show that a higher adsorption efficiency was obtained for the BC sample treated with a 1.0% NaOH solution. Therefore, this sample was used as adsorbent material in this work.

#### 3.2.1. Effect of Contact Time

**Figure 3:**
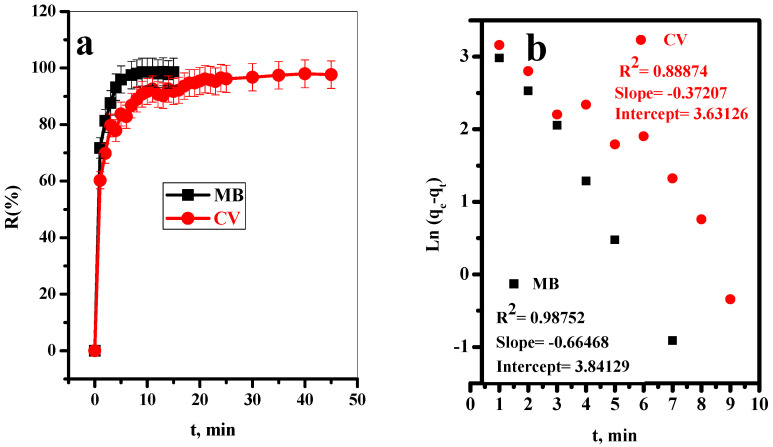

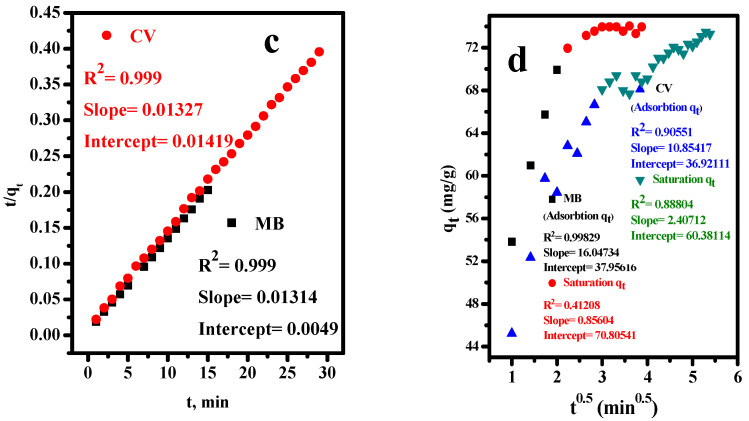
Effect of contact time on (**a**) removal percent, (**b**) pseudo first-order model, (**c**) pseudo second-order kinetic model, and (**d**) intra-partical diffusion models for adsorption of MB and CV-dye onto T-BC adsorbent (t = 15 min for MB and 45 min for CV, (dye) = 30 mg/L, dose = 20 mg, pH = 7, T = 25 °C).

The effect of contact time on the removal efficiency (%R) of MB and CV-dyes using BC treated with 1.0% NaOH aqueous solution (T-BC-1) from aqueous solution was presented in Figure 3a. It was observed that the adsorption percentages of both MB and CV-dyes increased rapidly in the first 15 min. This is may be due to the unique layered structure of the prepared T-BC, which will make a large number of surface-active sites of the adsorbent available for effective adsorption. After that, equilibrium was reached for both two dyes where the active sites of the adsorbent were fully occupied [48,49].

##### Adsorption Kinetics

The parameters obtained from the slope and intercept of the linear plots of pseudo-first-order, pseudo-second-order, and intra-particle diffusion models were calculated and represented in Figure 3b–d, respectively, and listed in Table 2. Based on the values obtained, the pseudo-second-order kinetic model was better suited to explaining the kinetics of MB and CV-dyes adsorption onto T-BC-sheets. The rate-limiting step could be chemical adsorption, where the adsorption capacities are proportional to the number of active sites occupied on the T-BC-surface [50], and the calculated values of q_e_ are 76.1035 and 75.3579 mgg^−1^ for MB and CV-dyes, respectively. These calculated values are closer to the experimental values of q_e exp,_ which are equal to 73.55 and 68.8008 mgg^−1^ for MB and CV-dyes, respectively. Furthermore, depending on the greater R^2^ of intra-particle diffusion, it was concluded that the intra-particle diffusion plays a considerable role in the adsorption of MB and CV species by T-BC. Nano-sheet diffusion and sorption into the interior are the two processes in the intra-particle diffusion process. The active centers on the surface of the modified biochar were approximately filled after 15 min for MB and 45 min for CV.

#### 3.2.2. Effect of Initial Concentration of Dyes

**Figure 4:**
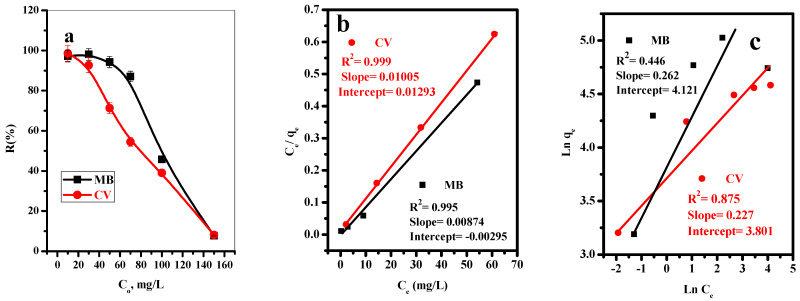
(**a**) Effect of initial dye concentration on adsorption percent of MB and CV, (**b**) Langmuir isotherm model, and (**c**) Freundlich isotherm model (t = 8 min for MB and 10 min for CV, [dye] = 10, 30, 50, 70, 100, 150 mg/L, dose = 20 mg, pH = 7, T = 25 °C).

The impact of the initial concentration of MB and CV-dyes on the adsorption efficiency was studied and plotted in Figure 4a. Figure 4a showed that as the initial concentration of MB and CV-dyes increases, the adsorption %R of MB and CV-dyes onto T-BC decreases. This is because the adsorbent had enough active sites at the low dye concentration because vacant active sites on the adsorbent surface were filled with MB and CV-dyes species, and saturation of the adsorbent surface was possible when the concentrations of the dye were increased. Saturation occurs due to a limited number of adsorbent surface sites. This refers to an increase in dye concentration; the number of the species of MB and CV-dyes in the solution will be exceeded over the number of active sites, leading to decreased removal efficiency.

#### 3.2.3. Adsorption Isotherm

Two isotherm models (Langmuir and Freundlich) were used to describe the interaction between the MB and CV-dyes molecules and T-BC. The Langmuir and Freundlich models’ linear relations were plotted, as shown in Figure 4b,c, and various parameters were calculated and summarized in Table 3. The Langmuir model is used to explain the adsorption of MB and CV dyes on the surface of T-BC based on the correlation coefficient (R^2^ = 0.996 for MB and 0.9998 for CV), which indicates the adsorption process happens on a homogeneous active site as a monolayer. Furthermore, the 1/n values for MB and CV-dyes are 0.2621 and 0.2273, respectively, indicating that the procedure is not conducive to adsorption [51,52].

#### 3.2.4. Effect of T-BC Adsorbent Dose

**Figure 5:**
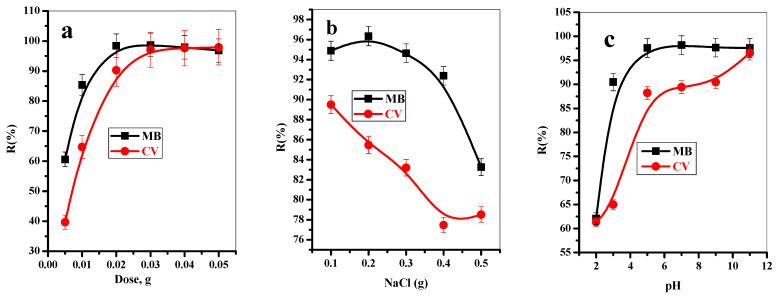
Effect of (**a**) adsorbent dose (t = 8 min for MB and 10 min for CV, [dye] = 30 mg/L, dose = 5, 10, 20, 30, 40, 50 mg, pH = 7, T = 25 °C), (**b**) NaCl dose (t = 8 min for MB and 10 min for CV, (dye) = 30 mg/L, dose = 20 mg, pH = 7, T = 25 °C, NaCL = 100–500 mg), and (**c**) pH on the removal percent of MB and CV-dyes from aqueous solution onto T-BC (t = 8 min for MB and 10 min for CV, (dye) = 30 mg/L, dose = 20 mg, pH = 2–11, T = 25 °C).

Figure 5a represents the influence of T-BC adsorbent dose on the adsorption percentage (%R) of MB and CV-dyes under the investigated conditions. The adsorption percentage of MB and CV-dyes increases with increasing the adsorbent dose in the range of 0.005–0.05 g at fixed dyes concentrations. It was observed that the removal efficiency of both MB and CV-dyes was enhanced with an increase in the adsorbent dose to 0.015 g for MB-dye and 0.025 g for the CV dye. This is attributed to the fact that with a further increase in the adsorbent dose, the surface area is enhanced, and the number of active sites increases, improving adsorption efficiency [3,53]. After that, the adsorption percent of both dyes kept constant with a further increase in the adsorbent dose. This may be due to a further increase in the adsorbent dose, as the adsorbent particles will be compacted together, and no active sites will be available for extra adsorption.

#### 3.2.5. Effect of NaCl Dose

For both dyes (MB and CV), different doses of sodium chloride in the range of 0.1–0.5 g were utilized to investigate the influence of salt concentration on %R of the investigated dyes onto TBC, as demonstrated in Figure 5b. It was observed that the removal percent of both dyes rapidly decreased with the addition of NaCl. This can be explained by the fact that, with increasing Cl^−^ concentrations, the competition for adsorption sites on T-BC adsorbent will increase and decrease analyte activity in media as the non-ideality of the solution increases. This could be because the presence of Na+ ion will block the active sites available for adsorption of the dye species, which leads to reducing the sorption percentage [3,53,54].

#### 3.2.6. Effect of pH

The initial pH of aqueous media affects the surface charges and dissociation of the functional groups of the adsorbents, as well as the degree of ionization of the adsorptive species, which has a considerable impact on the removal process. The adsorption of both MB and CV-dyes species was observed to be enhanced with a further increase in the pH from 2 to 7, after which the sorption percent was constant until pH equalled 11, as shown in Figure 5c. This is because, in an acidic environment, the high concentration of H-ion will compete with the dye species on the available active sites. Upon further increase in the pH value, the active sites will be more ionized, which will be suitable for efficient interaction with the MB and CV-dyes species; this behavior will enhance the adsorption performance [7,54,55].

#### 3.2.7. Effect of Temperature

**Figure 6:**
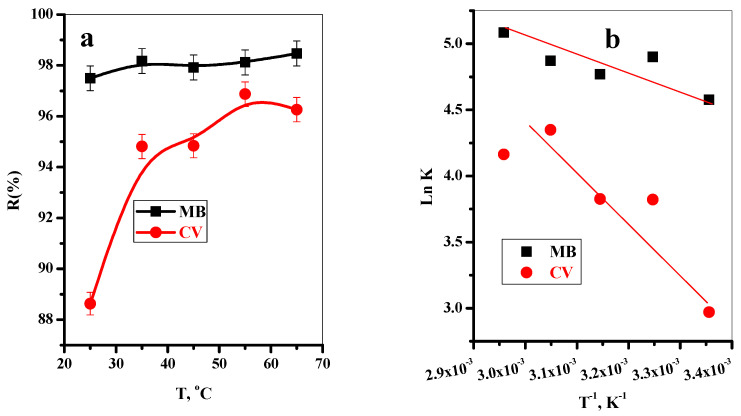
(**a**) Effect of temperature on the removal percentage of MB and CV dyes onto T-BC adsorbent, and (**b**) thermodynamic isotherm for the adsorption processes (t = 8 min for MB and 10 min for CV, (dye) = 30 mg/L, dose = 20 mg, pH = 7, T = 25 °C).

The impact of dye solution temperature in the range (25–65 °C) on the adsorption efficiency of MB and CV-dyes onto T-BC adsorbent was investigated, as shown in Figure 6a. The removal of MB-dye was slightly induced with the temperature, whereas the removal of CV-dye highly improved with an increase in the solution temperature. This may be described as follows: the dye solution temperature increases and the motion of the dye species increases, the collision between the dye species and adsorption sites increases, and the adsorption efficiency increases. Additionally, this could be due to a temperature-induced enhancement in porosity and total pore volume of the adsorbents [44,56].

#### 3.2.8. Thermodynamics Study

The thermodynamic studies were investigated to determine the thermal changes and spontaneous ability of the adsorption process. Table 4 includes the values of The Gibbs free energy change (∆G°) calculated from Equation S9 [57]. The entropy change (∆S°) and enthalpy change (∆H°) were obtained from the intercept and slope of the linear relation of ln K vs. 1/T (Figure 6b) and recorded in Table 4. The negative values of ∆G° cleared that the adsorption reaction of MB and CV is exergonic, and with the increase in the temperature, the negativity values increase, and the spontaneous ability of the reaction increase.

Moreover, the positive values of ∆H° indicate that the adsorption processes were endothermic in nature. This corresponded to energy absorption during MB and CV-species adhesion to the T-BC-surface. The positive values of ∆S° indicate that modified biochar had a considerable affinity for MB and CV-species. During the adsorption phase, it also demonstrated an increase in the unpredictability of the solid-solution interface [58].

### 3.3. Comparison of Maximum Adsorption Capacities with Different Natural Adsorbents

Table 5 illustrates the maximum adsorption capacities of MB-dye and CV-dye onto various types of natural biochars [1,22,24,28,33,59,60,61,62]. The results represented in this table shows that the fabricated T-BC have considerable adsorption capacities comparing with other published works. Additionally, T-BC can be regarded as a potential ecofriendly material to treat wastewaters with higher removal percentages for both dyes. From the data illustrated in Table 5, T-BC adsorbent shows ecofrindely, cost-effective, considerable removal ability, and other promising properties compared with various adsorbent materials.

## 4. Conclusions and Future Perspectives

This research demonstrates a practical method for producing effective biochar from agricultural wastes to remove dyes such as MB and CV-dyes from synthetic prepared wastewater. The adsorption capabilities of the prepared adsorbents were examined and compared. Overall, the dye adsorption capabilities of biochar treated with 1% NaOH were the best. Adsorption tests revealed that MB and CV-dyes had their highest adsorption capacity at a pH of 11, a dye concentration of 10 mg/L, and a temperature of 338 K. To characterize the adsorption behavior of MB and CV-dyes, the pseudo-second order kinetic and Langmuir models are sufficient. For the elimination of MB and CV-dyes using modified biochar, both endothermic and exothermic adsorptions were revealed. The removal of these organic molecules could be facilitated by electrostatic interactions, van der Waals forces, and hydrogen bonding. Finally, biochar made from agricultural wastes and treated with NaOH is a promising ecofriendly adsorbent for the treatment of wastewater and removal of organic pollutants. Additionally, further future works will be investigated to prepare other promising ecofriendly adsorbents by modification and treatment of sugarcane bagasse agricultural waste to acquire maximum benefit and to be applicable in various industrial applications. In terms of the outlook for the future, this research will contribute to the development of the preparation and applications of eco-friendly adsorbent materials from sugarcane bagasse agricultural waste.

## Figures and Tables

**Table 1:** The D and G-band shifts, FWHMs, and I_D_/I_G_ ratios.

Sample	G-Band		D-Band		I_D_/I_G_
	Raman Shift (cm^−1^)	FWHMs	Raman Shift (cm^−1^)	FWHMs	
SCB	1583	92	1353	157	1.71
BC	1581	92	1358	163	1.77
T-BC	1581	91	1330	175	1.92
T-BC-MB	1574	95	1377	182	1.91
T-BC-CV	1583	83	1364	65	0.78

**Table 2:** Adsorption kinetics and diffusion mechanism for removal of MB and CV-dyes.

Dye	q_e_ Exp (mg/g)	First-Order Kinetic Parameter			Second-Order Kinetic Parameter			Intra-Particle Diffusion					
		K_1_ (min^−1^)	q_ecal_ (mg/g)	R^2^	K_2_ (g/mg.min)	q_ecal_ (mg/g)	R^2^	Adsorption			Saturation		
								C	K	R^2^	C	K	R^2^
MB	73.55	−0.664	46.58	0.987	0.035	76.103	0.999	37.956	16.047	0.998	70.805	0.856	0.412
CV	68.80	−0.372	37.76	0.888	0.012	75.357	0.999	36.921	10.854	0.905	60.381	2.407	0.888

**Table 3:** Adsorption isotherms parameters for the sorption of MB and CV-dyes on modified biochar.

Dye	Langmuir Isotherm Model		Freundlich Isotherm Model		
	Q_o_ (mg/g)	R^2^	N	K_f_ (mg/g)	R^2^
MB	114.9425	0.99561	3.8155	61.6276	0.446
CV	99.5025	0.99979	4.4001	44.7772	0.87585

**Table 4:** Thermodynamic parameters for the sorption of MB and CV onto T-BC.

Dye	ΔH^°^ (kJ/mole)	ΔS^°^ (J/mole K^−1^)	ΔG^°^ (kJ/mol)				
			298 k	308 k	318 k	328 k	338 k
**MB**	8.2468	66.2328	−11.342	−12.548	−12.610	−13.287	−14.288
**CV**	24.8029	109.9622	−7.359	−9.785	−10.117	−11.859	−11.701

**Table 5:** Comparison of various adsorbents to adsorb MB and CV-dyes from aqueous media.

Adsorbent	MB					CV					Ref.
	Q_o_, mg g^−1^	Dosage (g/L)	Dye Conc. ppm,	pH	%R	Q_o_, mg g^−1^	Dosage (g/L)	Dye Conc. ppm,	pH	%R	
CNCs	94.43	10	5	8	85.7	∗	∗	∗	∗	∗	1
(Ec-bio)	123.3	0.3	50	10	79.2	56	0.4	40	4	87.11	22
Seaweed biochar	133.33	50	200	4	∗	∗	∗	∗	∗	∗	24
Biochars (BCs)	52.6	0.2	25	8–10	84.2	∗	∗	∗	∗	∗	28
(RH-MBC)	∗	∗	∗	∗	∗	80.04	5	100	∗	100	33
CNC/ZnO nanocomposite	64.93	4	100	4	97.5	∗	∗	∗	∗	∗	59
Wet-torrefied microalgal biochar	113.00	1	210	6	89.78	∗	∗	∗	∗	∗	60
GBC500	∗	∗	∗	∗	∗	23.71	2	5–200	8	∗	61
GBC300	∗	∗	∗	∗	∗	11.02	2	5–200	8	∗	61
Palm Kernel Shell-Derived Biochar	∗	∗	∗	∗	∗	24.45	16.7	400	7	86.4	62
T-BC	114.42	0.1	30	7	98.7	99.50	0.1	30	7	98	This work

∗ Not detected.

## Data Availability

Data are contained within the article.

## Supplementary Materials

The following supporting information can be downloaded at: https://www.mdpi.com/article/10.3390/polym14132587/s1, Figure S1: BET-Plot, BJH-Plot, Adsorption/desorption isotherm of SCB, BC and T-BC; Figure S2. EDX analysis of SCB, BC and T-BC; Figure S3. Effect of NaOH concentration on the adsorption efficiency of treated biochar; Table S1. Surface area, pore volume, and pore diameter of SCB, BC and T-BC. section S1.1. Materials and Instrumentation, S1.2. Characterization, S1.3. Adsorption experiment, S1.4. Adsorption kinetics, S1.5. Adsorption isotherm, S1.6. Thermodynamics study.

## Abbreviations

**Symbol**	**Nomenclature**
CV-dye	crystal violet
MB-dye	methylene blue
SCB	Sugarcane bagasse
(BC)	biochar
(Ec-bio)	eucalyptus camaldulensis biochar
(CNCs)	cellulose nanocrystals
(T-BC)	treated biochar
∆G°	energy change
∆S°	entropy change
∆H°	enthalpy change
RH-MBC	rice husk-mediated magnetic biochar
CNC/ZnO nanocomposite	cellulose nanocrystals incorporated with zinc oxide
GBC500	woody tree Gliricidia sepium at 500 °C
GBC300	woody tree Gliricidia sepium at 300 °C
