# Supplementary material for: Fabrication and Characterization of Effective Biochar Biosorbent Derived from Agricultural Waste to Remove Cationic Dyes from Wastewater
Source: Polymers (Basel). 2022 Jun 26;14(13):2587. doi: 10.3390/polym14132587 (PMC9269505; doi:10.3390/polym14132587)
Supplement: Supplementary file 1 [file polymers-14-02587-s001.zip › polymers-1763630-supplementary.pdf]

*Supplementary Materials*

# Fabrication and Characterization of Effective Biochar Biosorbent Derived from Agricultural Waste to Remove Cationic Dyes from Wastewater

**Asmaa Elsherbeny Moharm [1], Gamal A. El Naeem [2], Hesham M. A. Soliman [2], Ahmed I. Abd-Elhamid [2], Ali A. El-Bardan [1], Taher S. Kassem [1], AbdElAziz A. Nayl [3,*] and Stefan Bräse [4,5,*]**

[1] Department of Chemistry, Faculty of Science. Alexandria University, P.O. Box 426, Alexandria 21321, Egypt; asmaa.moharm1317@gmail.com (A.E.M.); alielbardan@yahoo.com (A.A.E.-B.); taherkasem@gmail.com (T.S.K.)
[2] Advanced Technology and New Materials Research Institute (ATNMRI), City of Scientific Research and Technological Applications (SRTA-City), New Borg AlArab, Alexandria 21934, Egypt; jimmynaeem@yahoo.co.uk (G.A.E.N.); h.soliman@srtacity.sci.eg (H.M.A.S.); ahm_ch_ibr@yahoo.com (A.I.A.-E.)
[3] Department of Chemistry, College of Science, Jouf University, Sakaka 72341, Saudi Arabia
[4] Institute of Organic Chemistry (IOC), Karlsruhe Institute of Technology (KIT), Fritz-Haber-Weg 6, 76133 Karlsruhe, Germany
[5] Institute of Biological and Chemical Systems-Functional Molecular Systems (IBCS-FMS), Director Hermann-von-Helmholtz-Platz 1, 76344 Eggenstein-Leopoldshafen, Germany
* Correspondence: aanayel@ju.edu.sa or aanayl@yahoo.com (A.A.N.); stefan.braese@kit.edu (S.B.)

## 1. Materials and Methods

### 1.1. Materials and Instrumentation

Distilled water, MB and CV (99.9%, Sigma Aldrich), SCB from a local market, NaOH , NaCl, and HCl (30%, El Salam for Chemical Industries). Water distillatory (2108, GLF, Germany) for double distilled water, pH meter (3510, Genway), centrifuge (Mikro 220R, Hettich, UK), analytical balance (CP 2245, Sartorius, USA), hot plate stirrer (SB 162, Stuart, UK), UV/Vis spectro-photometer (T80+, PG instruments Ltd., UK).

### 1.2. Characterization

Scanning electron microscope model (SEM, JEOL GSM-6610LV) and transition electron microscope model (TEM, JEOL GSM-6610LV, Japan) were used for investigating the surface morphology of resultant composite. Fourier transmission infrared spectroscopy (FT-IR) model (8400s Shimadzu, Japan) with a wavelength range from 4000 to 400 $cm^{-1}$ was used. Raman spectra were provided using Raman Microscope (Bruker, SenterraII, Germany) with an excitation wavelength of 514 nm and a power of 5 mW. Thermogravimetric analyzer model (Shimadzu thermal gravimetric analysis (TGA)—50, Japan) was investigated to detect the thermal stability of resultant composites. Thermal decomposition of samples was carried out under nitrogen with a heat rate of 10 °C/min.

### 1.3. Adsorption experiment

The MB and CV were dissolved in distilled water to make a 1000 ppm reserve solution, which was then diluted to various concentrations based on the experimental needs. The dye adsorption by produced materials was investigated using the color change of the MB and CV-dyes in an aqueous solution under continuous stirring. For both MB and CV, different dye concentrations (10, 30, 50, 70, 100, and 150 ppm) were used in a 100 ml glass beaker at different amounts of prepared materials (0.005-0.05 g), temperatures (25, 35, 45, 55, and 65 °C), pH (2, 3, 5, 7, 9, 11), and adding NaCl to the dye solution in various quantities was used to determine salinity in a range of concentrations (0.1-0.5g). Following that, 1.0 mL of the suspension was centrifuged. 1.0 ml of the solution was diluted after centrifugation to 5 ml with distilled water after phase separation. UV/Vis spectro-photometer (T80+, PG instruments Ltd., UK) was used to measure MB and CV concentrations at wavelengths of 662 nm and 590 nm, respectively. The optimum conditions reported in this investigation are, for MB-dye, (MB) = 30 ppm, dose = 0.02 g/50 ml, T = 25°C, pH = 7, and t = 15 min; for CV-dye, they are (CV) = 30 ppm, dose = 0.02 g/50 ml, T = 25°C, pH = 7, and t = 45 min, unless otherwise cited. Equation **(S1)** gives the dye removal efficiency (%R):

$$\%R = \frac{C_o - C_t}{C_o} \times 100 \quad \textbf{(S1)}$$

where $C_o$ and $C_t$ represent the initial and time-dependent dye concentrations, respectively.

**1.4. Adsorption kinetics**

The rate of adsorbate taken on by the adsorbent at constant pressure with respect to time is described by adsorption kinetics. The adsorption kinetics parameters computed are useful in anticipating the type of adsorption process that occurs by studying the adsorption rate over time. The experiment used kinetic models such as pseudo-first order and pseudo-second order. Lagergren equation Equation **(S2)** presents pseudo-first order:

$$\ln (q_e - q_t) = \ln q_e + K_{ads}\, t \quad \textbf{(S2)}$$

Where $q_e$ (mg /g) is the amount of sorption at equilibrium, $q_t$ (mg /g) is the amount of sorption over time, and $K_{ads}$ ($min^{-1}$) is the pseudo-first order sorption rate constant Equation **(S3)**:

$$q_e = \frac{(C_o - C_e)}{1000w} \times V \quad \textbf{(S3)}$$

where $C_o$ is the starting concentration (mg/L), $C_e$ stands for dye concentration at time intervals of equilibrium (mg/L), V is the dye solution volume (mL), and w is the adsorbent mass (g) Equation **(S4)**:

$$q_t = \frac{(C_o - C_t)}{1000w} \times V \quad \textbf{(S4)}$$

where $C_t$ represents the dye concentration over time (mg/L).

The Ho equation represents pseudo-second order. Equation **(S5)** represents the equation in a linear form:

$$\frac{t}{qt} = \frac{1}{K_2 \, q_e^2} + \frac{t}{q_e} \qquad \textbf{(S5)}$$

The rate constant of the reaction is $K_2$ (g/mg.min).

Equation **(S6)** was used to determine intra-particle diffusion:

$$\mathbf{qt = k_i \, t^{0.5} + c} \qquad \textbf{(S6)}$$

**1.5. Adsorption isotherm**

The adsorption isotherm was used to investigate the relationship between the amount of adsorbed adsorbate on the surface of the adsorbent and its quantity. The most well-known models for describing the adsorption isotherm are the Langmuir and Freundlich isotherms.

**1.5.1. Langmuir isotherm**

For monolayer adsorption of adsorbate species on equally energetic active sites of adsorbent, Equation **(S7)** gives the following model:

$$\frac{c_e}{q_e} = \left(\frac{1}{b\, q^\circ}\right) + \left(\frac{1}{q^\circ}\right) c_e \qquad \textbf{(S7)}$$

Where $C_e$ is the equilibrium concentration (mg/L), at equilibrium, $q_e$ is the amount adsorbed (mg/g), the maximum dye species uptake per unit mass of adsorbent is given by q° (mg/g), and b is the Langmuir constant (L/mol) **.**

**1.5.2. Freundlich isotherm**

The Freundlich model, often known as Freundek's theory, implies that the adsorbate species were adsorbed on the adsorbent's hetero-energetic active sites and is represented by Equation **(S8)**:

$$\ln Q_e = \ln K_F + \frac{1}{n} \ln C_e \qquad \textbf{(S8)}$$

Where $C_e$ denotes the equilibrium concentration in (mg/L), at equilibrium, Qe is the amount adsorbed (mg/g), and $K_f$ denotes the Freundlich adsorption equilibrium constant. In the Freundlich isotherm model, 1/n is another constant; when 1/n > 1, adsorption is advantageous, and when 1/n = 1, the process is homogenous. The procedure is not favorable to adsorption otherwise**.**

### 1.6. Thermodynamics study

The thermal changes and spontaneous adsorption process were calculated using thermodynamic analysis. The van 't Hoff plot, as depicted in Equation **(S9)**, described the relationship between temperature and the adsorption equilibrium constant. The Gibbs free energy change ($\Delta\mathbf{G}°$ in kJ mol$^{-1}$). The slope and intercept of the van 't Hoff plot of ln K vs 1/T were then used to derive the values of entropy change $\Delta\mathbf{S}°$ (kJ mol$^{-1}$) and enthalpy change $\Delta\mathbf{H}°$ (kJ mol$^{-1}$) Equations **(S10) and (S11)**. To determine whether the reaction between adsorbent and adsorbate is chemical or physical, endothermic or exothermic, and spontaneous or nonspontaneous, these parameters were calculated .

$$\mathbf{\Delta G° = -RT\ ln\ K} \quad \textbf{(S9)}$$

$$\boldsymbol{K} = \frac{\mathbf{q}_e}{\mathbf{c}_e}$$

T is the temperature (K), while the universal gas constant is R (8.314 J/mol K). Additionally,

$$\mathbf{\Delta G° = \Delta H° - T\Delta}\boldsymbol{S}° \quad \textbf{(S10)}$$

$$\mathbf{ln\ k} = \frac{\mathbf{\Delta S°}}{\mathbf{R}} - \frac{\mathbf{\Delta H°}}{\mathbf{RT}} \quad \textbf{(S11)}$$

## 2. Results and Discussion

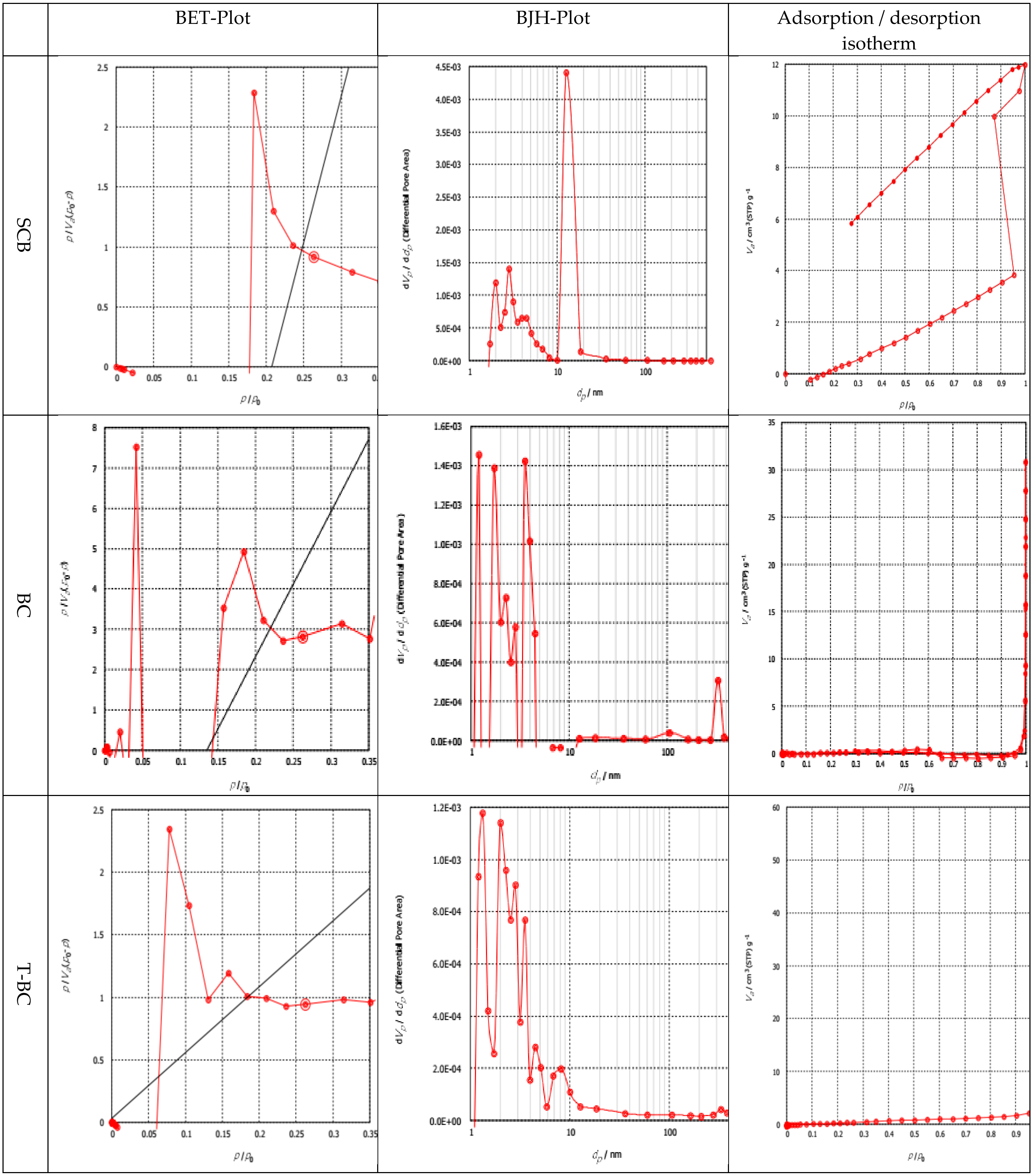


**Figure S1.** BET-Plot, BJH-Plot, adsorption / desorption isotherm of SCB, BC, and T-BC.

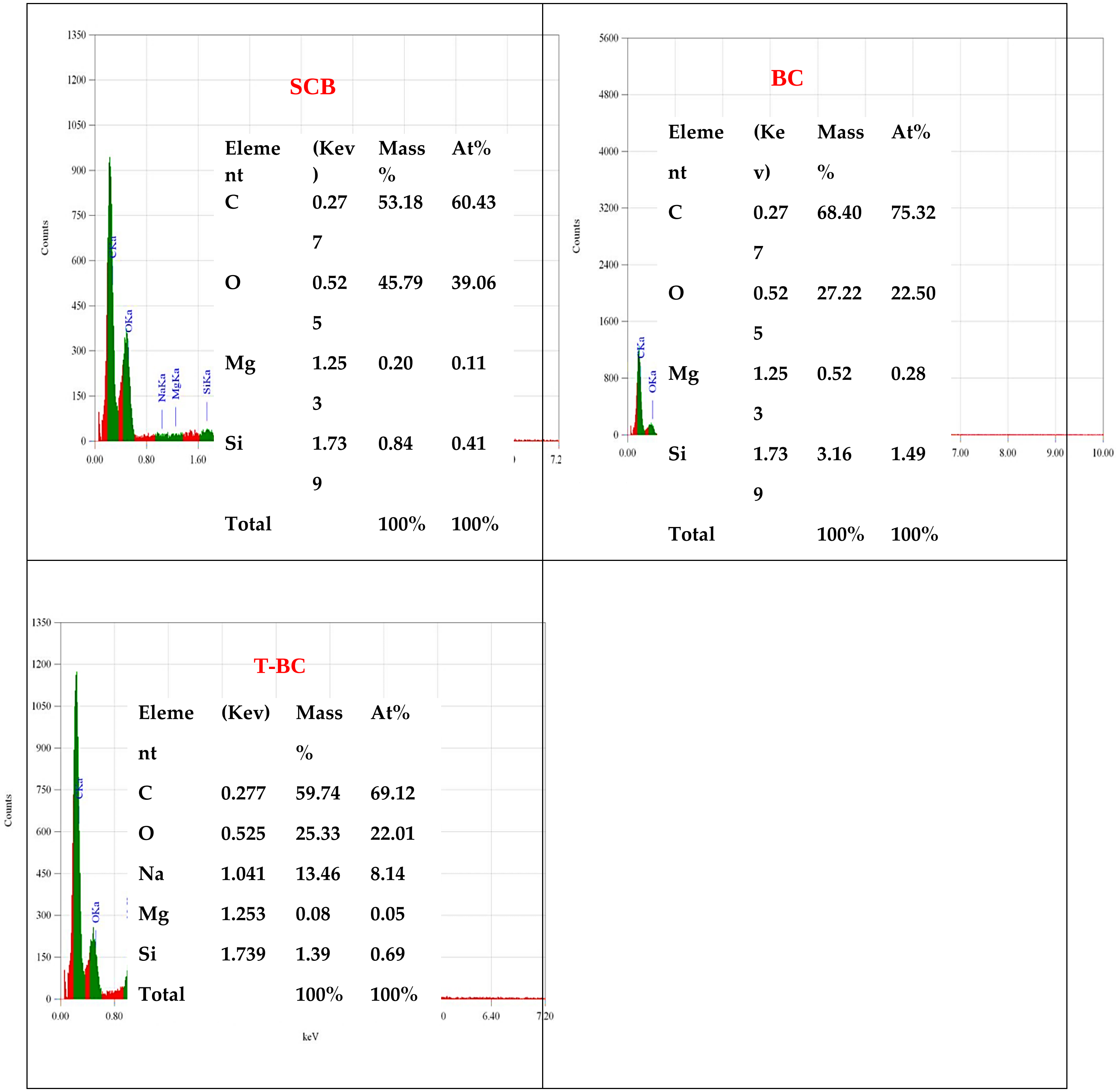


SCB

| Element | (Kev) | Mass % | At% |
|---|---|---|---|
| C | 0.277 | 53.18 | 60.43 |
| O | 0.525 | 45.79 | 39.06 |
| Mg | 1.253 | 0.20 | 0.11 |
| Si | 1.739 | 0.84 | 0.41 |
| Total | | 100% | 100% |

BC

| Element | (Kev) | Mass % | At% |
|---|---|---|---|
| C | 0.277 | 68.40 | 75.32 |
| O | 0.525 | 27.22 | 22.50 |
| Mg | 1.253 | 0.52 | 0.28 |
| Si | 1.739 | 3.16 | 1.49 |
| Total | | 100% | 100% |

T-BC

| Element | (Kev) | Mass % | At% |
|---|---|---|---|
| C | 0.277 | 59.74 | 69.12 |
| O | 0.525 | 25.33 | 22.01 |
| Na | 1.041 | 13.46 | 8.14 |
| Mg | 1.253 | 0.08 | 0.05 |
| Si | 1.739 | 1.39 | 0.69 |
| Total | | 100% | 100% |

**Figure S2.** EDX analysis of SCB, BC, and T-BC.

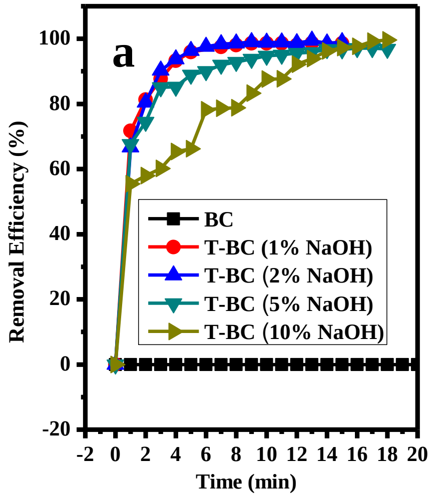


**Figure S3.** Effect of NaOH concentration on the adsorption efficiency of treated biochar.

**Table S1.** Surface area, pore volume, and pore diameter of SCB, BC, and T-BC.

| | **SCB** | | **BC** | | **T-BC** | |
|---|---|---|---|---|---|---|
| | **BET** | **BJH** | **BET** | **BJH** | **BET** | **BJH** |
| Surface area ($m^2/g$) | 2.2538 | 4.8861 | 1.4006 | 0.4389 | 8.2175 | 4.2079 |
| Total pore volume ($cm^3/g$) | 1.7911 | 1.9719 | 2.6560 | 1.4331 | 6.3102 | 1.7244 |
| Average pore diameter (nm) | 317.89 | 16.143 | 75.852 | 130.61 | 30.716 | 16.392 |
